# First person – Kun Guo

**DOI:** 10.1242/bio.058650

**Published:** 2021-03-26

## Abstract

First Person is a series of interviews with the first authors of a selection of papers published in Biology Open, helping early-career researchers promote themselves alongside their papers. Kun Guo is first author on ‘The thermal dependence and molecular basis of physiological color change in *Takydromus septentrionalis* (Lacertidae)’, published in BiO. Kun conducted the research described in this article while a PhD student in Xiang Ji's lab at Jiangsu Key Laboratory for Biodiversity and Biotechnology, College of Life Sciences, Nanjing Normal University, Jiangsu, China. They are now research assistant in the lab of Xiang Ji at College of Life and Environmental Sciences, Wenzhou University, Zhejiang, China, and Jiangsu Key Laboratory for Biodiversity and Biotechnology, College of Life Sciences, Nanjing Normal University, Nanjing, Jiangsu, China.


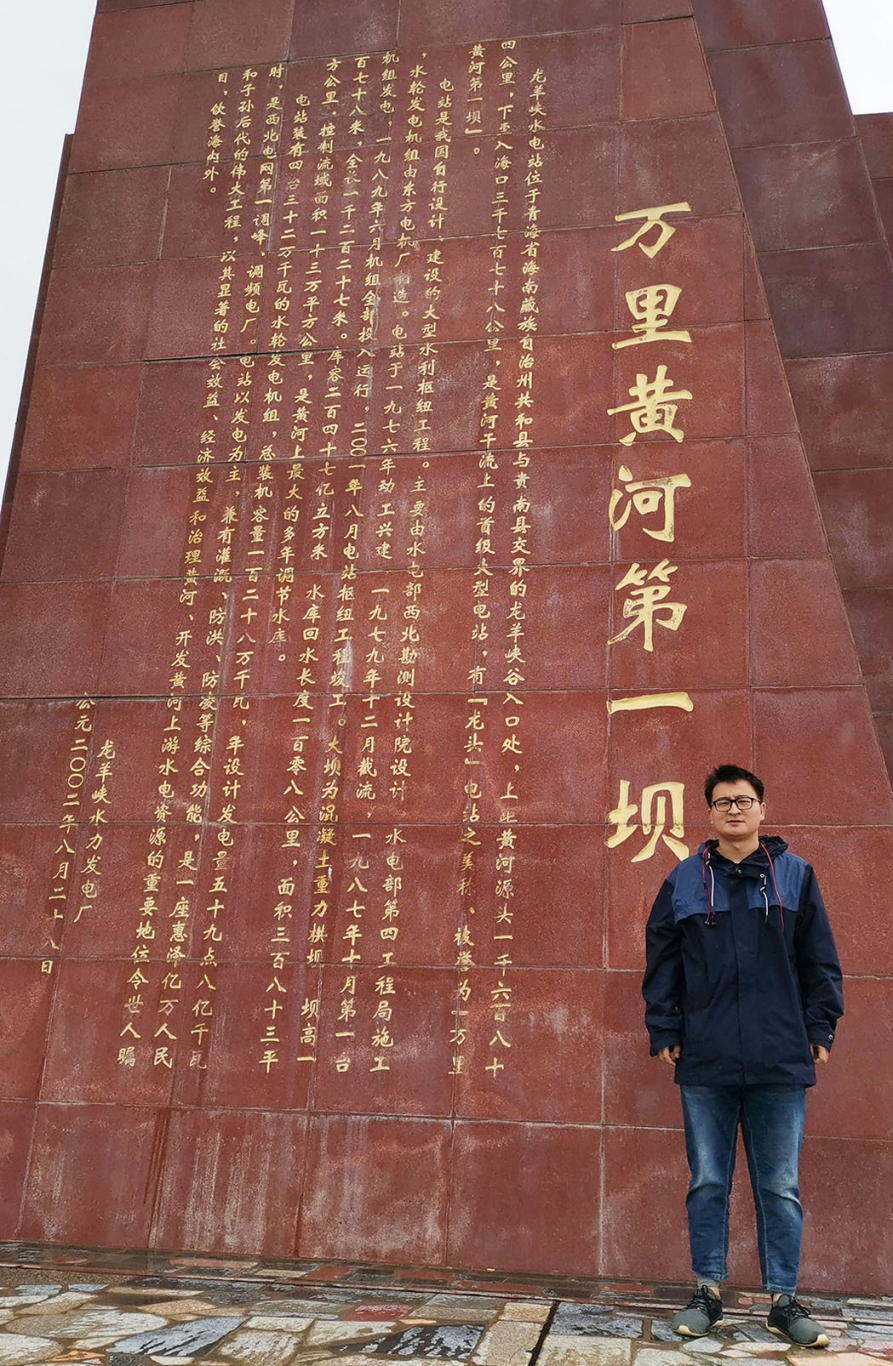


**Kun Guo**

**What is your scientific background and the general focus of your lab?**

Our scientific background is Kirchhoff's law of thermal radiation, which predicts absorption and release of radiation energy are positively correlated. Color change of lizards will be conducive to adapting themselves to the microenvironment. The lab where we did this work focuses on how amphibians and reptiles adapt to the environment, and its molecular mechanism. These opportunities helped me focus on hybrid speciation and adaptive evolution in reptiles under global warming.

“[I] focus on hybrid speciation and adaptive evolution in reptiles under global warming.”

**How would you explain the main findings of your paper to non-scientific family and friends?**

Temperature affects body color luminance, which generally decreases with increasing temperature at lower temperatures and increases with increasing temperature at higher temperatures. The lizards *T. septentrionalis* quickly change body color in response to the thermal environment around them, so that they could save a lot of time during thermoregulation. Furthermore, we explore the molecular mechanism of physiological color change found in *T. septentrionalis*, caused by the pigment aggregating quickly.

“The lizards *T. septentrionalis* quickly change body color in response to the thermal environment around them[.]”

**What are the potential implications of these results for your field of research?**

Our future work would usefully investigate whether the molecular basis of the thermal dependence of physiological color change observed in *T. septentrionalis* is generalizable to lizards. Therefore, we could evaluate stability and collapse of lizards under exposure to climate change.

**What has surprised you the most while conducting your research?**

The function curves of the thermal dependence of body color change is a U-shaped, which is a result of absorption and release of radiation energy.

**Figure:**
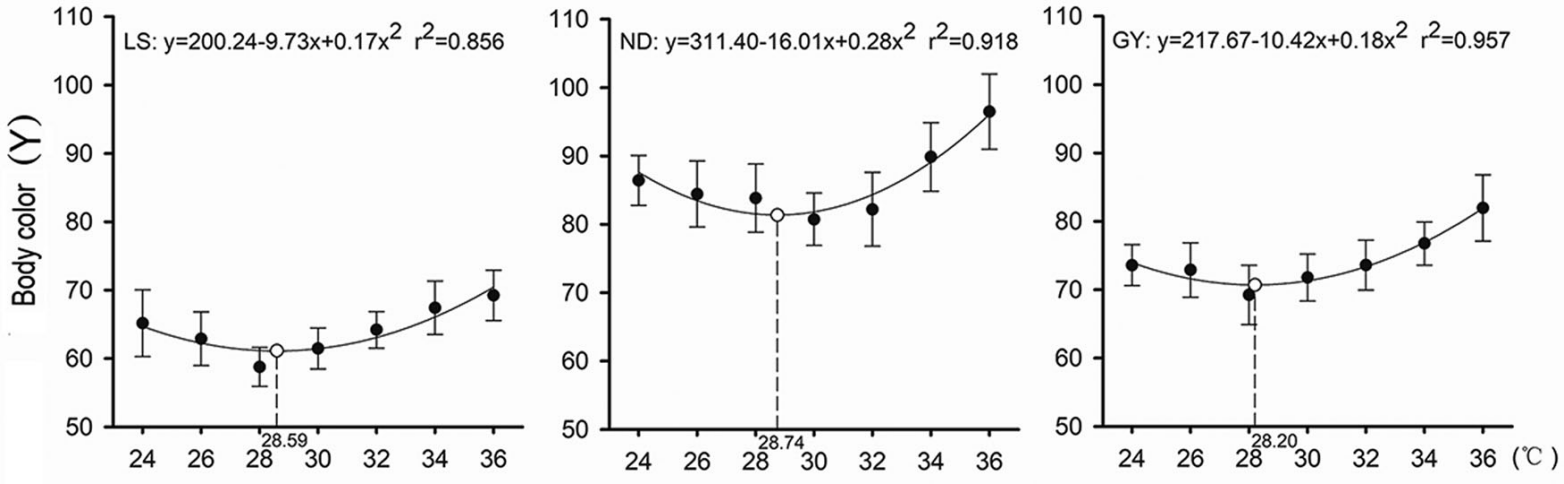
**The thermal dependence of body color change in *T. septentrionalis*.**

**What, in your opinion, are some of the greatest achievements in your field and how has this influenced your research?**

The color of animal variation along with latitudes and microenvironments are the greatest achievements in our field. This theory gives us an idea for adaptive evolution in lizards, which is the thermally sensitive animal. Therefore, we do some work in the thermal dependence of body color change to reveal its molecular mechanism.

**What changes do you think could improve the professional lives of early-career scientists?**

First, we should learn the related research literature as more as possible, and talk with the professor and learn from them. Then learning from young scientists is also helpful. And if possible, government aid for institutes to cover publishing or additional research costs.

**What's next for you?**

Our future work could usefully investigate whether the molecular basis of the thermal dependence of physiological color change observed in *T. septentrionalis* is generalizable to lizards. We will do the similar working in some species lizards, which are distribution in latitudes.
